# An Immune-Related Gene Signature Can Predict Clinical Outcomes and Immunotherapeutic Response in Oral Squamous Cell Carcinoma

**DOI:** 10.3389/fgene.2022.870133

**Published:** 2022-07-04

**Authors:** Liyuan Zhang, Xiaopeng Wang

**Affiliations:** Department of Oral and Maxillofacial Surgery, Qilu Hospital (Qingdao), Cheeloo College of Medicine, Shandong University, Qingdao, China

**Keywords:** oral squamous cell carcinoma, prognosis, tumor microenvironment, immunotherapeutic response, chemotherapy

## Abstract

**Objective:** Immune landscape is a key feature that affects cancer progression, survival, and treatment response. Herein, this study sought to comprehensively characterize the immune-related genes (IRGs) in oral squamous cell carcinoma (OSCC) and conduct an immune-related risk score (IRS) model for prognosis and therapeutic response prediction.

**Methods:** Transcriptome profiles and follow-up data of OSCC cohorts were curated from TCGA, GSE41613, and IMvigor210 datasets. An IRS model was established through univariate Cox, Random Survival Forest, and multivariate Cox analyses. Prognostic significance was evaluated with Kaplan–Meier curves, ROC, uni- and multivariate Cox, and subgroup analyses. A nomogram was conducted and assessed with C-index, ROC, calibration curves, and decision curve analyses. Immune cell infiltration and immune response were estimated with ESTIMATE and ssGSEA methods.

**Results:** An IRS model was constructed for predicting the overall survival and disease-free survival of OSCC, containing MASP1, HBEGF, CCL22, CTSG, LBP, and PLAU. High-risk patients displayed undesirable prognosis, and the predictive efficacy of this model was more accurate than conventional clinicopathological indicators. Multivariate Cox analyses demonstrated that this model was an independent risk factor. The nomogram combining IRS, stage, and age possessed high clinical application values. The IRS was positively associated with a nonflamed tumor microenvironment. Moreover, this signature enabled to predict immunotherapeutic response and sensitivity to chemotherapeutic agents (methotrexate and paclitaxel).

**Conclusion:** Collectively, our study developed a robust IRS model with machine learning method to stratify OSCC patients into subgroups with distinct prognosis and benefits from immunotherapy, which might assist identify biomarkers and targets for immunotherapeutic schemes.

## Introduction

Oral squamous cell carcinoma (OSCC) represents a heterogeneous malignancy arising from the mucosal lining of the oral cavity (Chai et al., 2020). This disease has a relatively low survival rate and an increasing incidence in some geographical areas (Almangush et al., 2020). The mainstays of OSCC therapy are surgery resection, chemotherapy, radiotherapy, or a combination of these modalities, which depend on the degree of this disease and patients’ comorbid factors (Chai et al., 2020). At present, prognostic biomarkers of OSCC patients mainly depend on the TNM staging system. However, the system cannot be insufficiently and inaccurately predictive of patients’ survival rate because the prognosis of patients in the same category varies greatly (Lydiatt et al., 2017). Although immune checkpoint inhibitors have been proven for improving prognosis of patients with recurrent or metastatic OSCC, there is still a lack of reliable biomarkers to stratify OSCC patients and predict therapeutic responses (Kenison et al., 2021). So far, the immune background and clinical implication of OSCC remain uncertain.

Tumorigenesis is a complex process, which involves interactions between tumor cells, tumor microenvironment, and immune system, affecting tumor onset and progression (Niu et al., 2022). The complex interplay of tumor cells with the immune system facilitates tumor immune evasion, eventually leading to tumor growth, metastasis, and treatment failure (Chen L. et al., 2021). Accumulating evidence demonstrates that immune cells and immune-related genes (IRGs) in the tumor microenvironment exert essential accessory roles in prognosis, progression, resistance, and immunotherapeutic responses of OSCC patients (Kondoh et al., 2019). For instance, high CXCL14 expression contributes to decreased tumor growth and enhanced lymphocyte infiltration in OSCC (Parikh et al., 2020). Hence, in-depth characterization of immune landscape in OSCC may facilitate to uncover the critical functions of immune surveillance and evasion triggering OSCC initiation and progression as well as offer necessary evidence for informing rational treatment design and predicting therapeutic responses and patients’ survival outcomes.

## Materials and Methods

### Curation of OSCC Cohorts and Data Preprocessing

Level 3 RNA sequencing (RNA-seq) data and clinical features of 328 OSCC samples were curated from The Cancer Genome Atlas (TCGA) *via* the Genomic Data Commons (GDC) Data Portal (https://portal.gdc.cancer.gov/) utilizing TCGAbiolinks package (Colaprico et al., 2016). Raw expression data (fragments per kilobase million (FPKM)) were processed into Transcripts Per Kilobase Million (TPM). Microarray expression profiling and clinical information of 97 OSCC patients were retrieved from the GSE41613 dataset through the Gene Expression Omnibus (GEO) repository (https://www.ncbi.nlm.nih.gov/geo/) based on the GPL570 platform (Lohavanichbutr et al., 2013). The raw “CEL” file of microarrays was standardized with robust multiarray averaging method utilizing affy (Gautier et al., 2004) and simpleaffy packages (Wilson and Miller, 2005). Supplementary Table S1 listed the patient demographics of the TCGA and GSE41613 datasets. 2,498 immune-related genes (IRGs) were curated from the Immunology Database and Analysis Portal (IMMPORT; https://www.immport.org) (Bhattacharya et al., 2018).

### Establishment and Verification of an Immune-Related Risk Score

Univariate cox regression models were conducted for the evaluation of the associations of IRGs with OSCC prognosis in the TCGA dataset. IRGs with *p* < 0.05 were significantly associated with the OSCC prognosis. The Random Survival Forest algorithm was adopted for ranking the relative importance of prognostic IRGs (Wang and Zhou, 2017). The number of Monte Carlo iterations was set as 100, and the number of steps forward was 5. The IRGs whose relative importance as characteristic genes were >0.4. Afterwards, a multivariate cox regression model was conducted. The calculation formula of the IRS model was as follows: $\mathrm{risk}\;\mathrm{score=}\overset{n}{\underset{k-1}{\sum }}Exp_{k}*e^{HR}k$ , where N represented the number of characteristic IRGs, Exp_k_ represented the expression level of characteristic IRGs, and e^HR^k meant the regression coefficient of genes. Based on the median value of IRS, OSCC patients in the TCGA or GSE41613 cohort were separated into high- and low-risk groups. Kaplan–Meier (K-M) curves with the log-rank test were adopted for assessing the statistical significance of overall survival (OS) and disease-free survival (DFS) rates between groups utilizing the survival package. Receiver operating characteristic (ROC) curves at 1-, 3-, and 5-year OS or DFS were conducted through the survivalROC package. Meanwhile, the area under the curve (AUC) was determined at each time-point for evaluating the discrimination (Heagerty et al., 2000). In the GSE41613 dataset, prognostic significance of each characteristic IRG was assessed with the K–M curves.

### Assessment of Predictive Performance of IRS

Through ROC curves, the predictive efficacy in the OSCC prognosis of IRS was compared with clinicopathological factors in the TCGA dataset. Uni- and multivariate cox regression models were conducted for evaluation of the predictive independency of IRS and clinicopathological factors in the OSCC prognosis. TCGA OSCC patients were separated into distinct subgroups according to clinicopathological factors, and the K–M curves of OS were conducted between high- and low-risk patients.

### Construction and Evaluation of a Prognostic Nomogram

A nomogram comprising IRS and clinicopathological features was established with the rms package. The Harrell’s concordance index (C-index) was calculated for assessing the performance of the constructed nomogram and each variable in this nomogram. Discrimination was verified through ROC curves at 1-, 3-, and 5-year follow-up. Prediction accuracy was evaluated via comparison of predicted and actual survival by calibration plots. Moreover, the decision curve analysis (DCA) was utilized for examining the clinical utility of this model via quantification of the net benefit at distinct threshold probabilities.

### Genetic Mutation Analysis

Somatic variant profiles of 508 OSCC patients stored in the mutation annotation format (MAF) were curated from the TCGA project, which were analyzed with the maftools package (Mayakonda et al., 2018). The tumor mutation burden (TMB) score was evaluated for each OSCC patient (Robinson et al., 2017). According to whether the top five genes were mutated or not, OSCC patients in the TCGA dataset were separated into mutated and nonmutated subgroups. In each subgroup, the K–M curves of OS were carried out between high- and low-risk patients.

### Function Enrichment Analysis

After separating TCGA OSCC patients into high- and low-risk groups, Kyoto Encyclopedia of Genes and Genomes (KEGG) pathways underlying the IRS were investigated through the Gene set enrichment analysis (GSEA) software (Subramanian et al., 2005). The “c2. cp.kegg.v6.1. symbols” from the Molecular Signatures Database (MSigDB; http://www.broadinstitute.org/msigdb) acted as the reference set (Liberzon et al., 2015). The number of random sample permutations was set at 1,000. The 50 hallmark gene sets were also collected from the MSigDB. The Single-Sample Gene Set Enrichment Analysis (ssGSEA) function of the Gene-set variation analysis (GSVA) (Hänzelmann et al., 2013) was utilized for estimating the enrichment score of hallmark pathways.

### Analysis of Immune Cell Infiltration

The estimation of stromal and immune cells in malignant tumors using the Expression data (ESTIMATE) (Yoshihara et al., 2013) was utilized for inferring the fractions of stromal and immune cells, and stromal score, immune score, tumor purity, and ESTIMATE score were determined, respectively. Through the ssGSEA method, the relative abundance of 29 immune signatures was quantified across OSCC specimens based on 29 published immune-related genes (comprising immune cell types, functions, and pathways).

### Analysis of Immunotherapeutic Responses

Immunotherapeutic responses were evaluated according to human leukocyte antigen (HLA), immune checkpoints, Tumor Immune Dysfunction and Exclusion (TIDE), and cancer immunity cycle. TIDE was scored based on two mechanisms of tumor immune evasion: inducing T-cell dysfunction in tumors with enhanced infiltrations of cytotoxic T lymphocytes (CTLs) and preventing T-cell infiltrations in tumors with reduced infiltrations of CTLs (Jiang et al., 2018). The cancer immunity cycle may reflect the antitumor immune response (Chen and Mellman, 2013). The activity of each step was quantified with the ssGSEA method based on the gene expression profiles of OSCC specimens.

### Collection of an Immunotherapeutic Cohort

Transcriptome profiles and follow-up information of patients with advanced urothelial cancer who received anti-PD-L1 therapy were curated from the IMvigor210 cohort (Mariathasan et al., 2018). Following the normalization, with the same formula, the IRS of each patient was calculated and the K–M curves of OS were conducted between high- and low-risk groups. The predictive efficacy of IRS was verified through the ROC curves.

### Analysis of Sensitivity to Anticancer Drugs

Through establishing the ridge regression model on the basis of Genomics of Drug Sensitivity in Cancer (GDSC; www.cancerrxgene.org/) (Yang et al., 2013), The pRRophetic package was adopted for predicting the half-maximal inhibitory concentration (IC50) of OSCC patients in the TCGA cohort (Geeleher et al., 2014). The IC50 values of anticancer drugs were utilized for inferring the drug sensitivity.

### Statistical Analysis

All data were analyzed with the R software, version 3.6.3. Survival curves were conducted with the K–M method, and the log-rank test was adopted for estimating the statistical significance. Comparisons between groups were presented with the Wilcoxon rank-sum test. Associations of variables were determined with the Spearman coefficients. The statistical test was two-sided, and the significance level was set at *p* < 0.05.

## Results

### Construction of an IRS Model for Prediction of OS and DFS of OSCC

**FIGURE 1:**
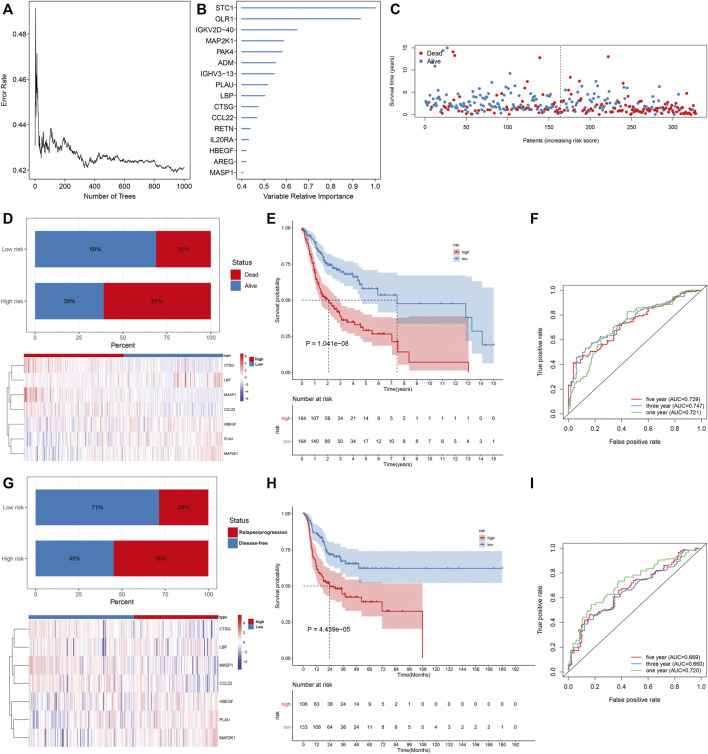
Construction of an IRS model for prediction of OS and DFS of OSCC in the TCGA cohort. **(A)** Associations of error rate with number of classification trees. **(B)** Relative importance ranking of 15 out-of-bag genes. **(C)** Visualization of survival time of each OSCC patient. **(D)** Distribution of survival status and expression patterns of characteristic IRGs in high- and low-risk groups. **(E)** The K–M curves of OS between two groups. **(F)** ROC curves at 1-, 3-, and 5-year OS based on the IRS. **(G)** Distribution of disease-free status and expression patterns of characteristic IRGs in high- and low-risk groups. **(H)** The K–M curves of DFS between two groups. **(I)** ROC curves at 1-, 3- and 5-year DFS based on IRS.

This study collected IRGs from the IMMPORT project. Univariate cox regression analysis uncovered that 172 IRGs were significantly associated with the OSCC prognosis, of which 86 were protective factors while 86 were risk factors (Supplementary Table S2). Through the Random Survival Forest algorithm, feature selection was presented. The relationships of error rate with number of taxonomic trees were utilized for revealing IRGs with relative importance >0.4 as characteristic genes (Figure 1A). Finally, the characteristic IRGs were screened, and the relative importance ranking of the out-of-bag scores for the characteristic IRGs are shown in Figure 1B. Through the multivariate cox regression analysis, an IRS model was established in the line with the following formula: risk score = MASP1 expression * (−0.31342) + HBEGF expression * 0.206009 + CCL22 expression * (−0.20744) + CTSG expression * (−0.29364) + LBP expression * 0.417348 + PLAU expression * 0.1248 + MAP2K1 expression * 0.442468 (Table 1). OSCC patients in the TCGA cohort were stratified into high- and low-risk groups at the median cut-off of IRS. In Figure 1C, we observed the prominently shorter survival time in high- than low-risk groups. There was higher percentage of dead patients in high-risk groupscompared with low-risk groups (61% vs. 31%; Figure 1D). Meanwhile, CTSG, LBP, MASP1, and CCL22 expressions displayed marked upregulation, while HBEGF, PLAU, and MAP2K1 expressions exhibited prominent downregulation in high-risk groupdthan in low-risk groups (Figure 1D). Low-risk patients displayed marked advantage in OS than those with high-risk (Figure 1E). The AUCs at 1-, 3-, and 5-year OS were separately 0.721, 0.747, and 0.729, indicative of the sensitivity and accuracy of IRS in the prediction of OS (Figure 1F). In Figure 1G, we noticed a lower percentage of disease-free patients in high-risk groups compared with low-risk groups (45% vs. 71%). Heat map depicted the prominent heterogeneity in the expression of the characteristic IRGs between the two groups. Compared with the low-risk group, markedly unfavorable DFS was investigated in the high-risk group (Figure 1H). The AUCs at 1-, 3-, and 5-year DFS were 0.720, 0.660, and 0.669, respectively, indicating that IRS possessed the potential in predicting the OSCC recurrence or progression (Figure 1I).

**TABLE 1:** Multivariate cox regression analysis of characteristic IRGs among OSCC patients in the TCGA cohort.

IRGs	Coefficient	HR	HR.95L	HR.95H	*p* value
MASP1	−0.31342	0.730943	0.600768	0.889324	0.001736
HBEGF	0.206009	1.228765	1.038503	1.453883	0.016389
CCL22	−0.20744	0.812666	0.691191	0.955489	0.012033
CTSG	−0.29364	0.745543	0.615232	0.903456	0.002738
LBP	0.417348	1.51793	1.276406	1.805155	2.36E-06
PLAU	0.1248	1.132921	0.964931	1.330158	0.127503
MAP2K1	0.442468	1.556544	1.156869	2.0943	0.003474

### External Verification of Predictive Performance of IRS in OSCC Prognosis

**FIGURE 2:**
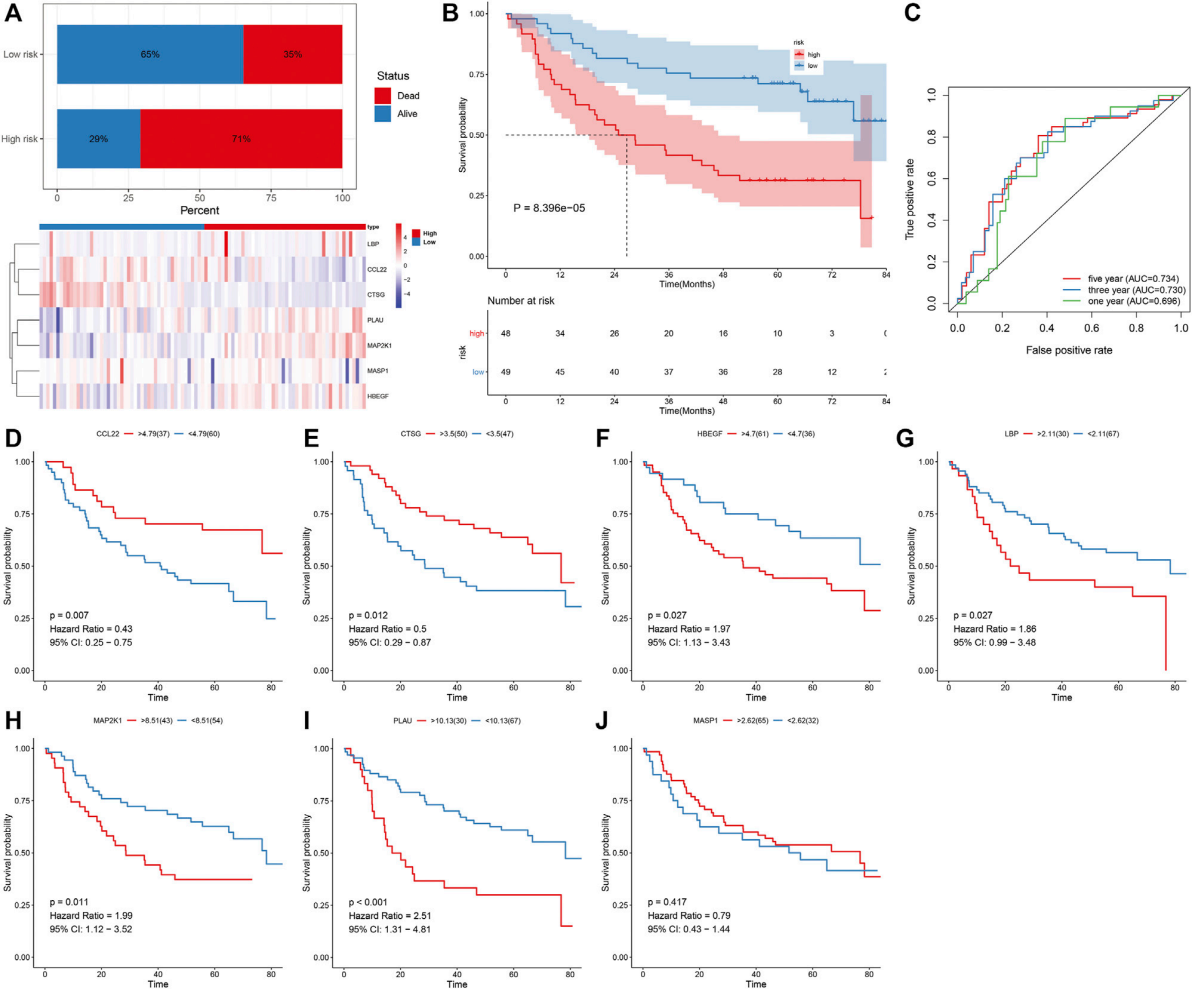
External verification of predictive performance of IRS in OSCC prognosis in the GSE41613 cohort. **(A)** Distribution of survival status and expression patterns of characteristic IRGs in high- and low-risk groups. **(B)** The K–M curves of OS between two groups. **(C)** ROC curves at 1-, 3-, and 5-year OS based on the IRS. **(D–J)** The K–M curves of OS between high and low expression of **(D)** CCL22, **(E)** CTSG, **(F)** HBEGF, **(G)** LBP, **(H)** MAP2K1, **(I)** PLAU, and **(J)** MASP1 groups.

The predictive efficacy of IRS was externally verified in the prediction of OSCC survival outcomes in the GSE41613 cohort. With the same formula, we determined the IRS of each patient. As expected, the increased percentage of dead patients was investigated in high-risk groupsthan in low-risk groups (Figure 2A; 71% vs. 35%). The prominent heterogeneity in the expression of the characteristic IRGs was confirmed between groups. In Figure 2B, high-risk patients displayed more undesirable OS in comparison with those with low-risk. The AUCs at 1-, 3-, and 5-year OS were separately 0.696, 0.730, and 0.734 (Figure 2C), confirming the favorable performance in the predicting OS. We also evaluated the prognostic significance of each characteristic IRG in OSCC patients. The upregulation of CCL22 and CTSG expressions indicated more desirable OS (Figures 2D,E), while high expressions of HBEGF, LBP, MAP2K1, and PLAU were in relation to more unfavorable OS (Figures 2F–I). Nevertheless, MASP1 did not markedly affect OS of OSCC patients (Figure 2J).

### Assessment of the Reliability and Independency of IRS in OSCC Prognosis

**FIGURE 3:**
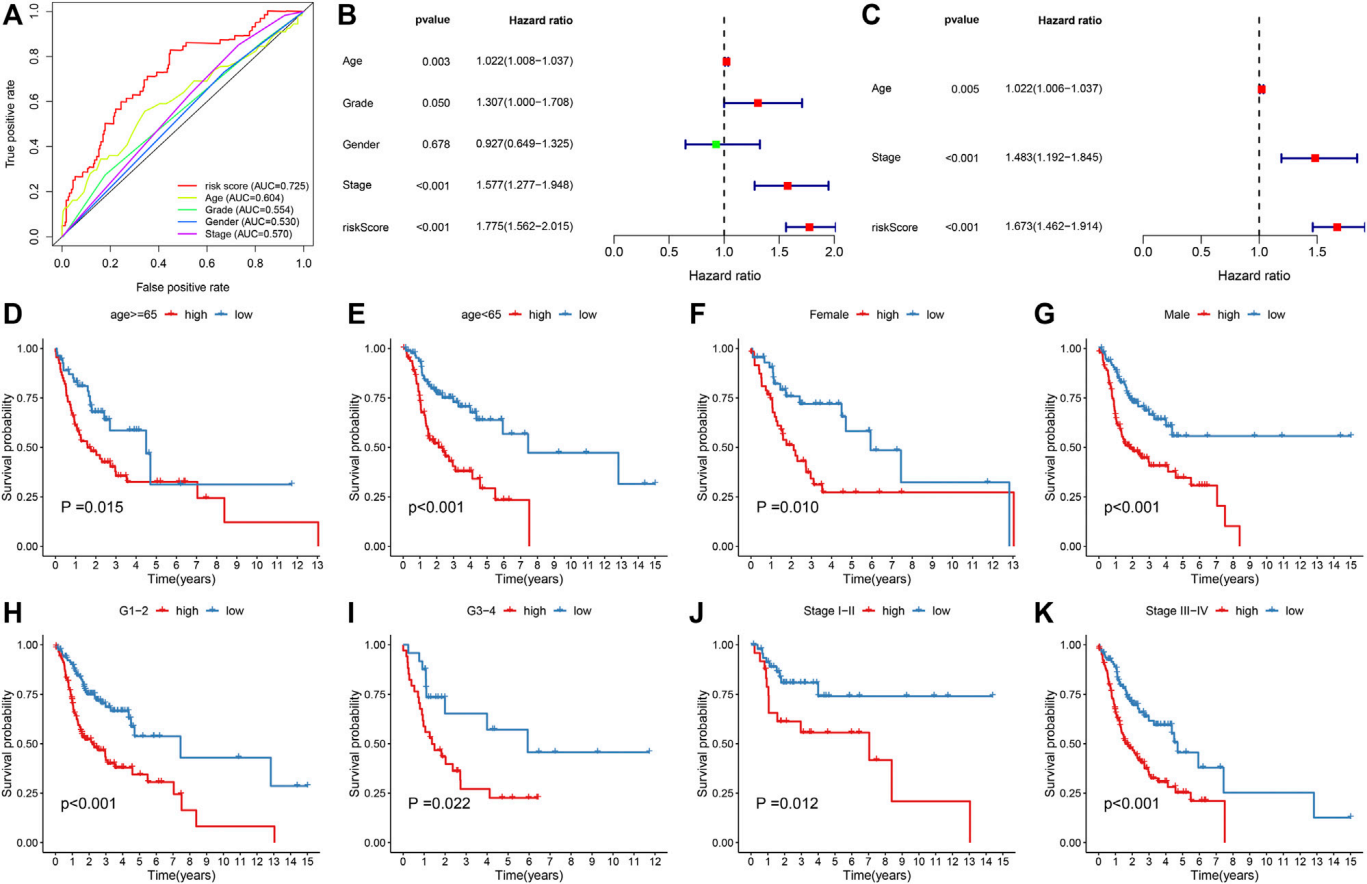
Assessment of the reliability and independency of IRS in OSCC prognosis in the TCGA cohort. **(A)** Comparison of the predictive efficacy of the IRS with clinicopathological factors via ROC curves. **(B,C)** Uni- and multivariate cox regression models for the associations of IRS and clinicopathological factors with OSCC prognosis. **(D–K)** The K–M curves of OS between high- and low-risk patients in each subgroup, including age ≥65 and <65; female and male; G1-2 and G3-4; stage I-II and stage III-IV.

We compared the predictive efficacy of IRS with clinicopathological factors via ROC curves. Our results demonstrated that the IRS possessed the highest AUC value, indicated that the IRS was superior to conventional clinical features in predicting the prognosis of OSCC (Figure 3A). Through uni- and multivariate cox regression models, IRS, stage, and age acted as independent risk factors of OSCC prognosis (Figures 3B,C). Subgroup analysis was presented for the assessment of the predictive sensitivity of IRS in OSCC survival outcomes. According to clinicopathological factors, OSCC patients were stratified into distinct subgroups, including age ≥65 and <65; female and male; G1-2 and G3-4; stage I-II and stage III-IV. We noticed that high-risk patients possessed poorer OS than those with low-risk in each subgroup (Figures 3D–K).

### Development of a Prognostic Nomogram for OSCC

**FIGURE 4:**
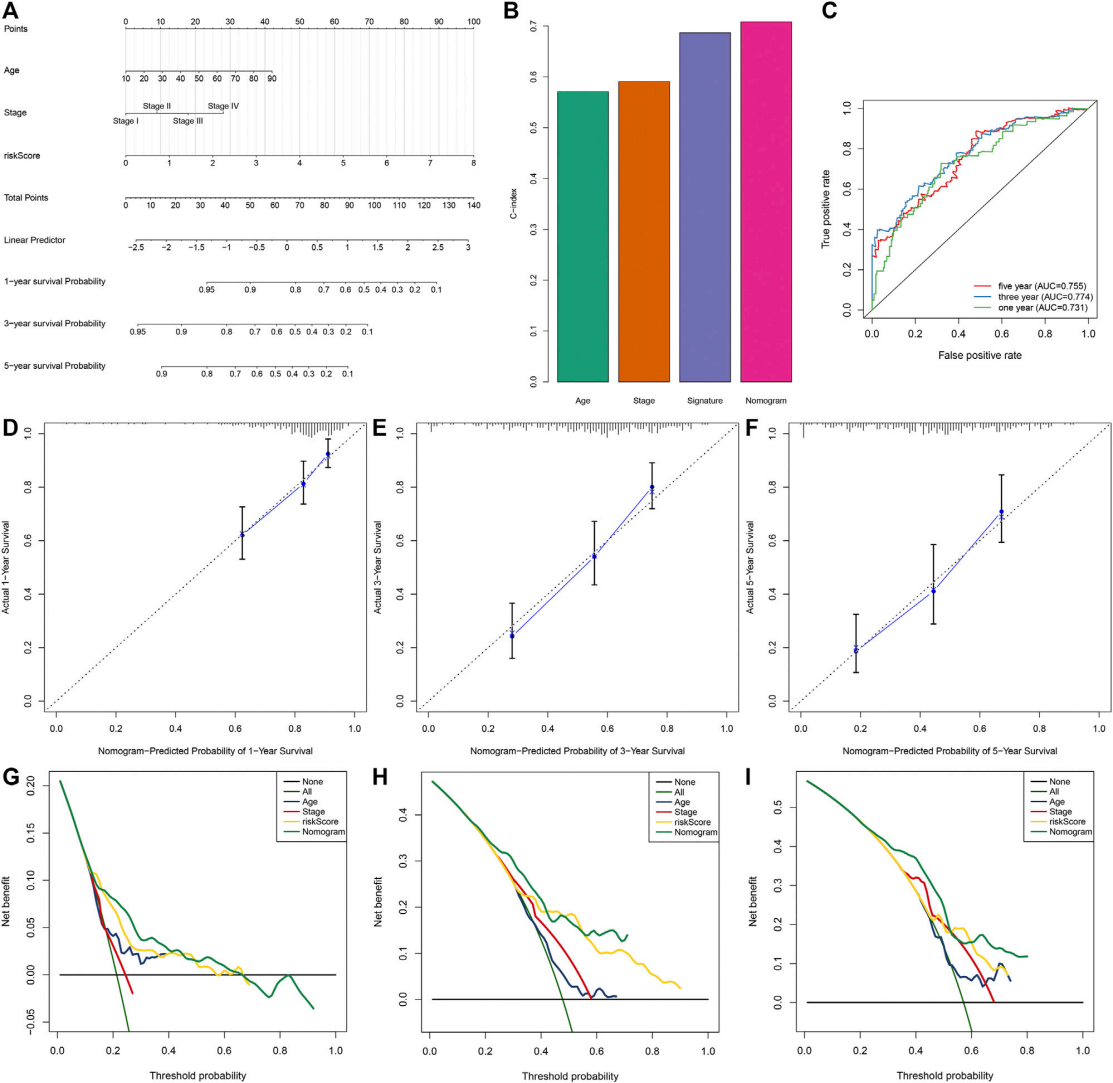
Development of a prognostic nomogram for OSCC patients in the TCGA dataset. **(A)** The nomogram covering IRS, stage, and age for prediction of 1-, 3-, and 5-year survival probabilities. **(B)** C-index of age, stage, IRS, and nomogram in predicting OS. **(C)** ROC curves at 1-, 3-, and 5-year OS based on this nomogram. **(D–F)** Calibration curves of this nomogram in prediction of 1-, 3-, and 5-year OS. **(G–I)** DCA of nomogram, age, stage, and IRS in terms of OS of OSCC patients. *X*-axis indicated the threshold probability, and *Y*-axis demonstrated the net benefit.

A prognostic nomogram derived from independent risk variables (IRS, stage, and age) was conducted, which enabled the determination of each patient’s score of each variable and the estimation of the survival probability (Figure 4A). C-index was calculated for assessing the predictive ability. The nomogram displayed more favorable accuracy in predicting OS prediction with a higher C-index compared with IRS, stage, and age (Figure 4B). Moreover, the AUCs at 1-, 3-, and 5-year OS were 0.731, 0.774, and 0.755, indicative of the favorable predictive performance of the nomogram (Figure 4C). Calibration curves for the probabilities of 1-, 3-, and 5-year survivals were indicative of the desirable agreement between the nomogram prediction and actual outcomes (Figures 4D–F). In DCA, the constructed nomogram exhibited a higher net benefit together with a broader range of threshold probabilities in comparison with age, stage, and IRS (Figures 4G–I). Therefore, this nomogram exhibited powerful predictive capacity for the prognosis of OSCC patients.

### IRS Model Is Independent of Genetic Mutations in Predicting Prognosis

**FIGURE 5:**
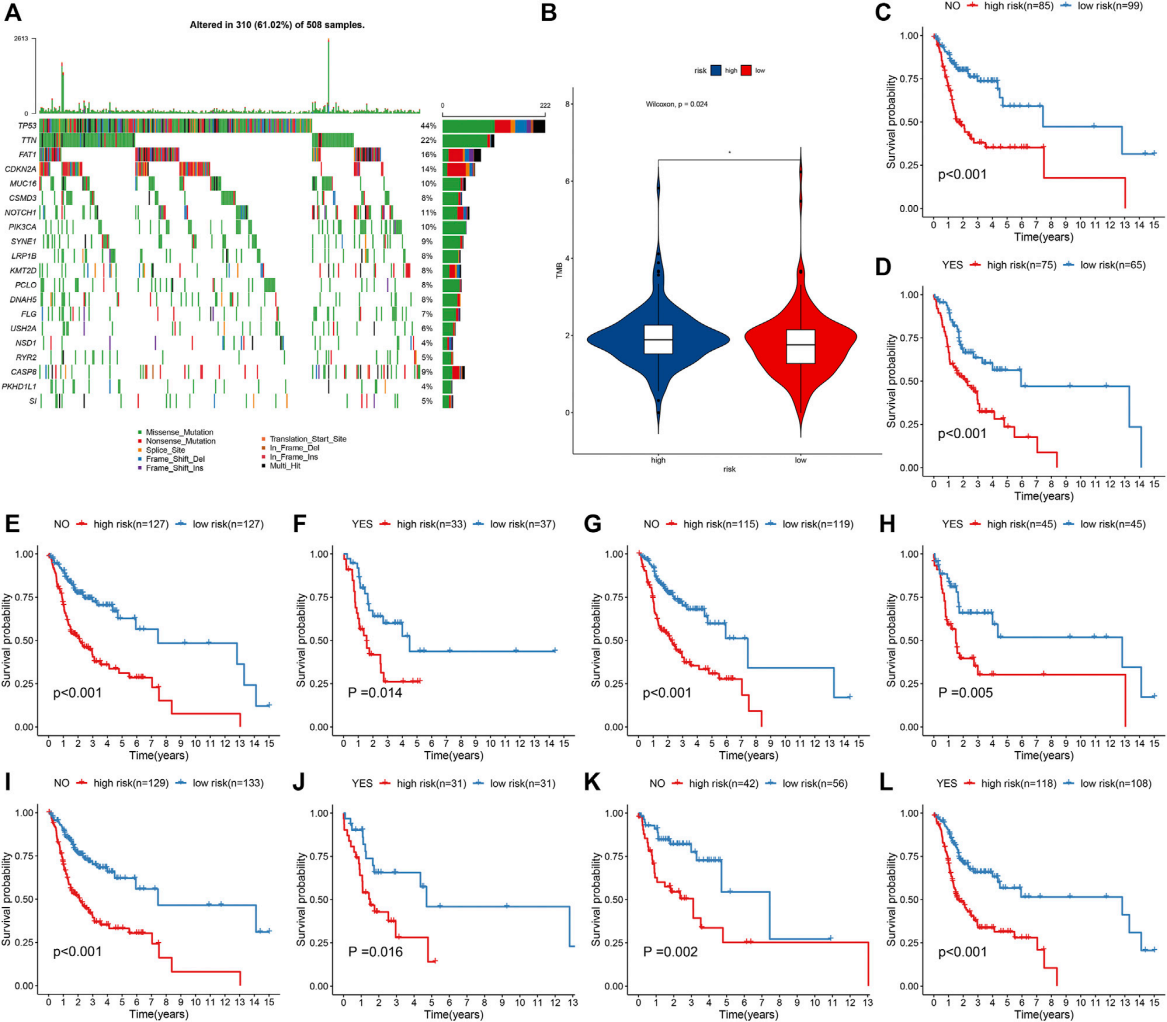
IRS model is independent of genetic mutations in predicting prognosis of OSCC patients from the TCGA cohort. **(A)** Waterfall plots showing the mutation distributions of the first 20 most frequently mutated genes. The upper panel showed the mutation frequency in each OSCC specimen. The central panel depicted the mutation types across OSCC patients. The bar plots on the right side showed the frequency and mutation types of genes. The bottom panel meant the legends for mutation types. **(B)** Comparison of TMB score in high- and low-risk groups. *p < 0.05. **(C–L)** K–M curves of OS between high- and low-risk patients in each subgroup, including mutated and nonmutated TTN, CDKN2A, FAT1, MUC16, and TP53.

Across 508 OSCC samples, 310 occurred somatic mutations (61.02%). The first 20 mutated genes are shown in Figure 5A. According to the mutation frequency, TP53 (44%), TTN (22%), FAT1 (16%), CDKN2A (14%), and MUC16 (10%) were the top five mutation genes. Compared with the high-risk group, significantly a reduced TMB score was found in the low-risk group (Figure 5B). OSCC patients were stratified into distinct subgroups according to mutated or nonmutated TTN, CDKN2A, FAT1, MUC16, and TP53. We found that high IRS was indicative of worse OS than low IRS in each subgroup (Figures 5C–L). Thus, the IRS model could be independent of genetic mutations in predicting the prognosis of OSCC patients.

### Biological Phenotypes Underlying IRS Model

**FIGURE 6:**
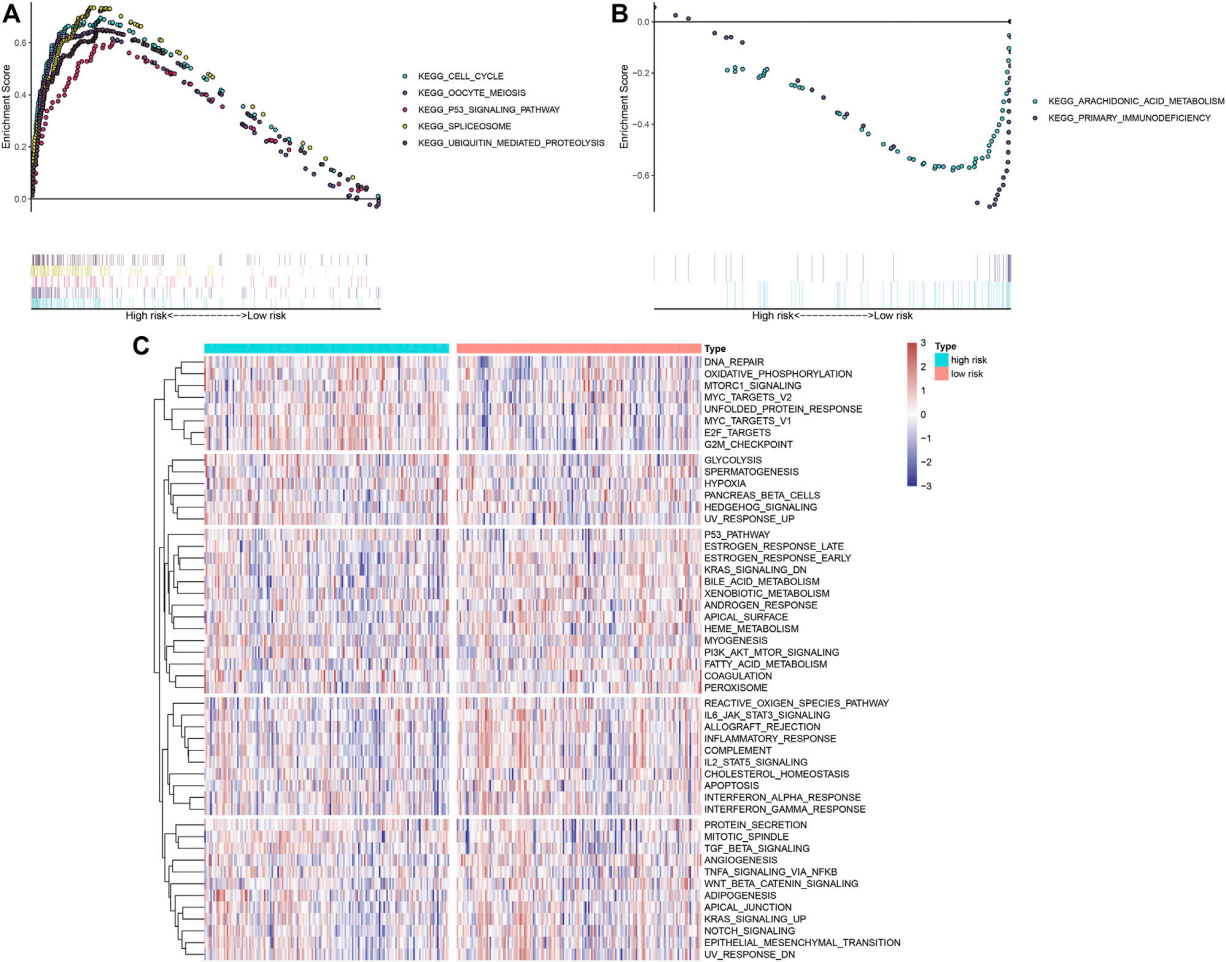
Biological functions involving the IRS model. **(A,B)** GSEA delineated biological pathways associated with the IRS model based on “c2. cp.kegg.v6.1. symbols” gene set. OSCC specimens in the TCGA dataset were separated into high- and low-risk groups. Each run was presented with 1,000 permutations. Enriched pathways with prominent correlations between two groups were separately depicted. **(C)** Heat map showing the activity of hallmark pathways in high- and low-risk groups.

GSEA uncovered that the cell cycle, oocyte meiosis, p53 signaling pathway, spliceosome, and ubiquitin-mediated proteolysis were markedly activated in high-risk OSCC specimens (Figure 6A). Meanwhile, arachidonic acid metabolism and primary immunodeficiency were significantly activated in low-risk OSCC specimens (Figure 6B). Through the ssGSEA method, we noticed the prominent activity of immune activation pathways such as IL6-JAK-STAT3 signaling, allograft rejection, inflammatory response, complement, and IL2-STAT5 signaling in the low-risk group (Figure 6C). Moreover, carcinogenic activation pathways like TGF-beta signaling, PI3K-Akt-mTOR signaling, E2F targets, MYC targets, and mTORC1 signaling displayed increased activity in the high-risk group.

### High IRS Is in Relation to a Nonflamed Tumor Microenvironment

**FIGURE 7:**
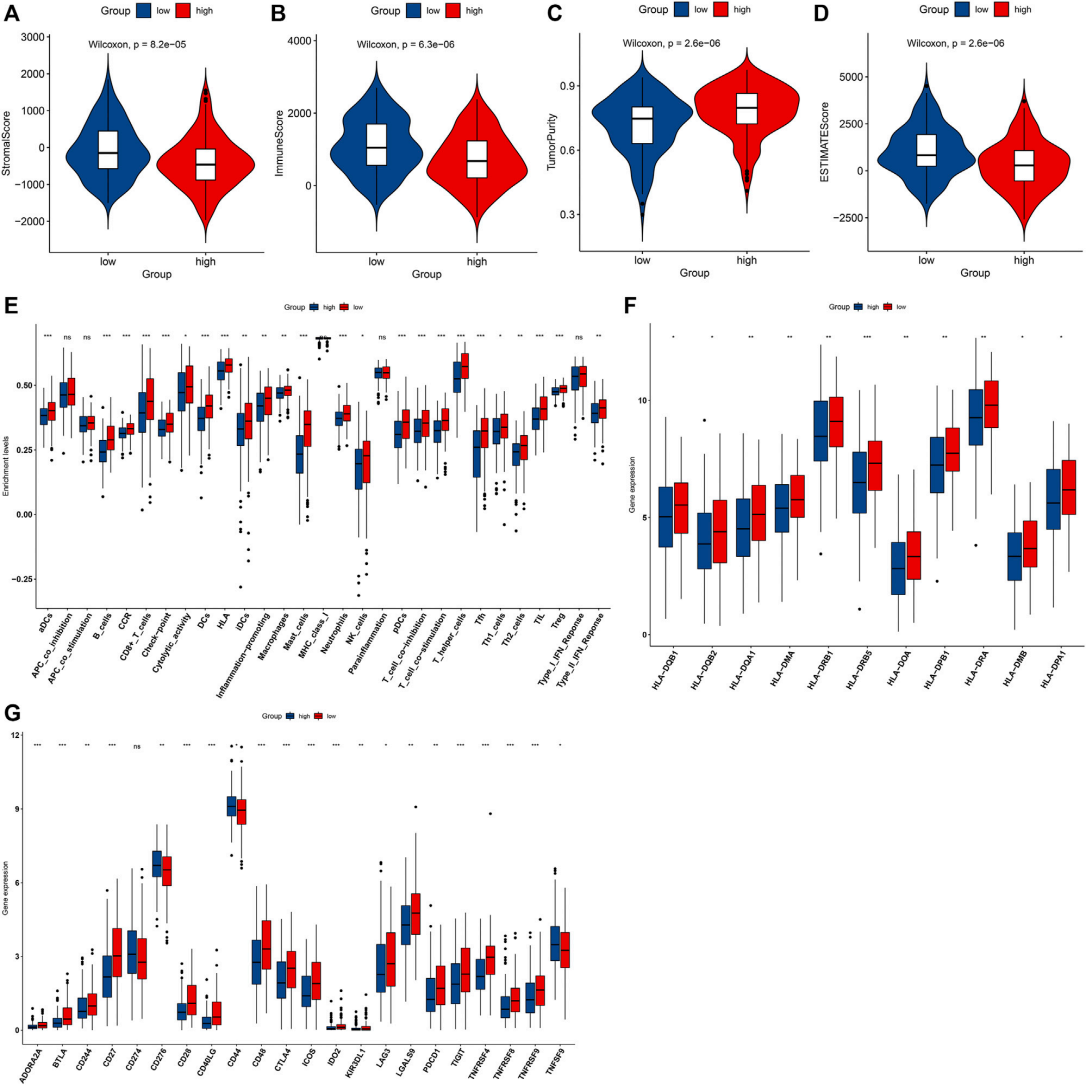
High IRS is in relation to a nonflamed tumor microenvironment in OSCC specimens from the TCGA dataset. **(A–D)** Comparisons of stromal score, immune score, tumor purity, and ESTIMATE score in high- and low-risk groups. **(E)** Comparisons of the abundance levels of immune cell types, functions, and pathways in high- and low-risk groups. **(F,G)** Comparisons of mRNA expression of HLA genes and immune checkpoints in two groups. Ns: not significant; *p < 0.05; **p < 0.01; and ***p < 0.001.

Through the ESTIMATE method, we investigated that high-risk OSCC specimens exhibited reduced stromal score, immune score, and ESTIMATE score but increased tumor purity in comparison with low-risk specimens (Figures 7A–D). In Figure 7E, low-risk OSCC displayed significant increase in aDCs, CD8^+^ T cells, DCs, HLA, iDCs, inflammation-promoting, macrophages, mast cells, MHC class I, neutrophils, NK cells, pDCs, and type II IFN response. Moreover, we investigated the prominent increase in the mRNA expression of HLA genes including HLA-DQB1, HLA-DQB2, HLA-DQA1, HLA-DMA, HLA-DRB1, HLA-DRB5, HLA-DOA, HLA-DPB1, HLA-DRA, HLA-DMB, and HLA-DPA1 in low-risk OSCC specimens (Figure 7F). In Figure 7G, most of immune checkpoints exhibited prominent upregulation in low-risk patients, including ADORA2A, BTLA, CD244, CD27, CD28, CD40LG, CD48, CTLA4, ICOS, LAG3, LGALS9, PDCD1, TIGIT, TNFRSF4, TNFRSF8, and TNFRSF9. This indicated that high IRS was in relation to a nonflamed tumor microenvironment of OSCC.

### IRS Predicts Immunotherapeutic Response

**FIGURE 8:**
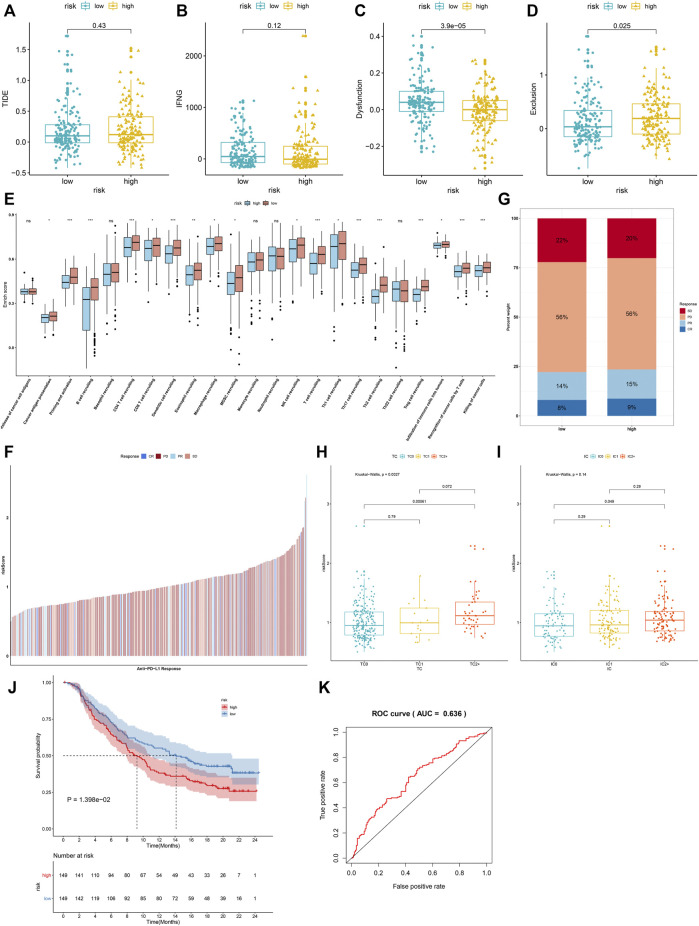
IRS predicts immunotherapeutic response. **(A–D)** Comparisons of TIDE score, T-cell receptor interferon-gamma (IFN-G), T-cell dysfunction, and T-cell exclusion in high- and low-risk groups. **(E)** Differences in each step of the cancer immunity cycle in high- and low-risk groups. **(F)** Distribution of IRS among patients who had different responses (CR, complete response; PR, partial response; PD, progressive disease; SD, stable disease) to anti-PD-L1 therapy in the IMvigor210 cohort. **(G)** Percentage of different responses to anti-PD-L1 therapy in high- and low-risk groups. **(H)** Comparisons of IRS in diverse tumor cell populations. **(I)** Comparisons of IRS in diverse tumor cell types. **(J)** The K–M survival curves between high- and low-risk patients treated with anti-PD-L1 therapy. **(K)** ROC curves of survival based on IRS. Ns: not significant; *p < 0.05; **p < 0.01; and ***p < 0.001.

We investigated the increased TIDE score and T-cell exclusion as well as the reduced IFNG and T-cell dysfunction in high-risk groupsthan in low-risk groups (Figures 8A–D). Moreover, there were reduced activities of nearly all steps in the cancer immunity cycle (including cancer antigen presentation, priming and activation, B-cell recruiting, CD4^+^ T-cell recruiting, CD8^+^ T-cell recruiting, dendritic cell recruiting, eosinophil recruiting, macrophage recruiting, MDSC recruiting, NK cell recruiting, T-cell recruiting, Th1 cell recruiting, Th17 cell recruiting, Th2 cell recruiting, Treg cell recruiting, infiltration of immune cells into tumors, recognition of cancer cells by T cells, and killing of cancer cells) in high-risk groupscompared with low-risk groups (Figure 8E). In the IMvigor210 cohort, relatively higher clinical responses to anti-PD-L1 therapy were found in high-risk patients (Figures 8F,G). Moreover, the IRS was markedly reduced in a deserted phenotype, TC0 (tumor cells with the lowest PD-L1 expression) and IC0 (immune cells with the lowest PD-L1 expression), as shown in Figures 8H,I. A survival analysis was then presented for assessing whether the IRS could predict the survival outcomes of patients treated with anti-PD-L1 therapy. High IRS was indicative of more undesirable clinical outcomes (Figure 8J). The AUC was 0.636, indicative of the wonderful performance in predicting patients’ prognosis (Figure 8K). Thus, IRS possessed the potential in predicting the immunotherapeutic response.

### IRS Predicts Anticancer Drug Responses of OSCC Patients

**FIGURE 9:**
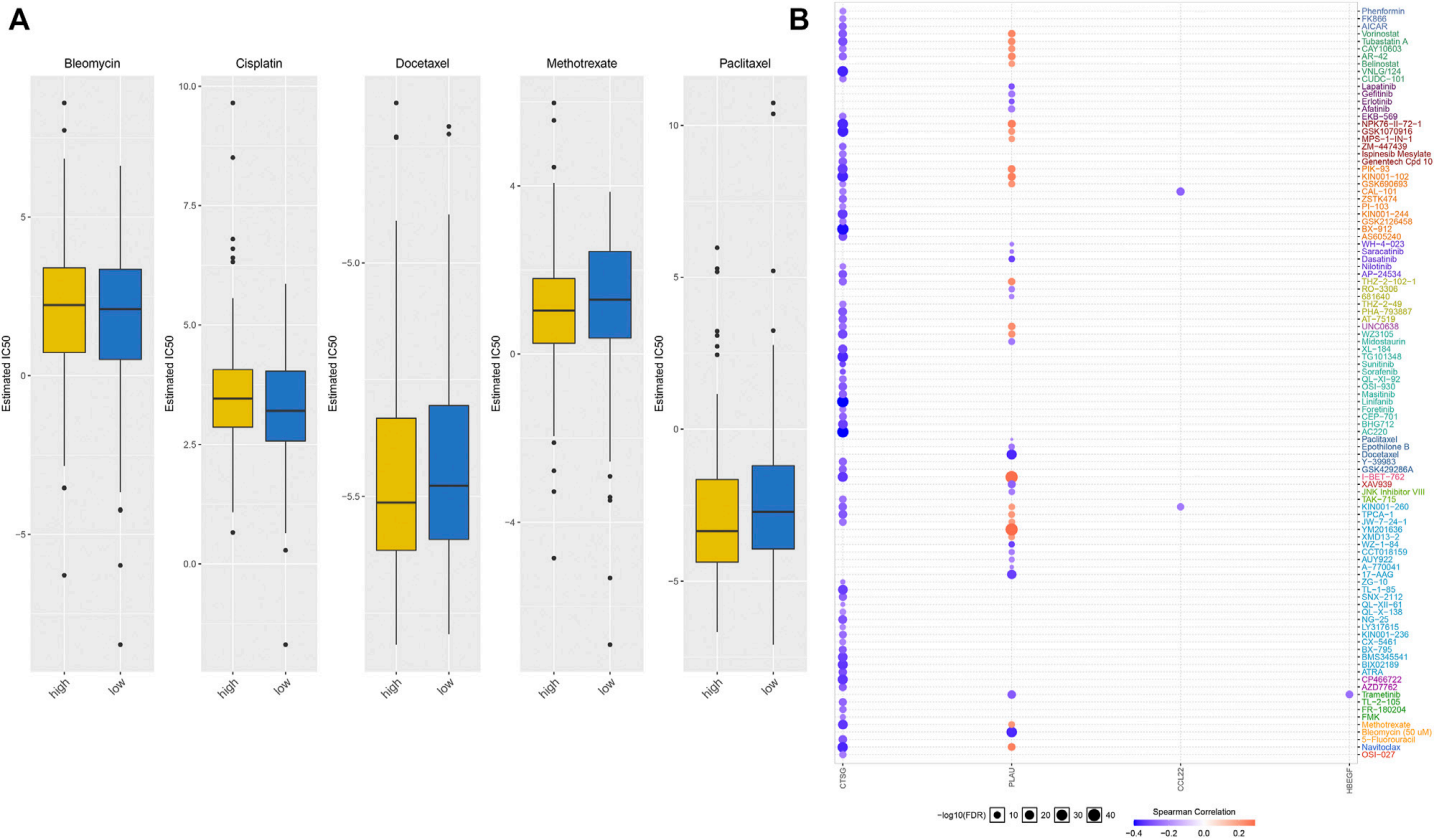
IRS predicts anticancer drug responses of OSCC patients. **(A)** Comparisons of IC50 values of chemotherapeutic agents in high- and low-risk groups. **(B)** Spearman correlation analysis of the correlations of each characteristic IRG with IC50 values of anticancer drugs.

We evaluated the differences in IC50 values of common chemotherapeutic agents (bleomycin, cisplatin, docetaxel, methotrexate, and paclitaxel) between high- and low-risk groups. As a result, the high-risk group had markedly reduced IC50 values of methotrexate and paclitaxel than low-risk group (Figure 9A). This indicated that high-risk patients displayed higher responses to methotrexate and paclitaxel. We also evaluated the associations of each characteristic IRG with IC50 values of anticancer drugs across OSCC patients. We observed that CTSG expression was negatively correlated to IC50 values of anticancer drugs, while PLAU expression displayed positive correlations to most anticancer drugs (Figure 9B). This indicated that IRS predicted anticancer drug responses of OSCC patients.

## Discussion

OSCC occupies about 90% of all oral cancer cases, which is characterized by high metastasis and recurrence rates and undesirable survival outcomes (Diao et al., 2021). Hence, it is crucial to determine specific and effective markers to forecast theOSCC prognosis. Evidence suggests that complicated alterations in IRGs contribute to the occurrence and progression of OSCC (Zhao et al., 2021). Nonetheless, critical IRG signatures and their prognostic value in OSCC require further exploration, which could deepen our comprehending of OSCC and assist determine certain patients who may benefit from immunotherapy. This study conducted an IRS model (containing MASP1, HBEGF, CCL22, CTSG, LBP, and PLAU) for the prediction of OS and DFS of OSCC patients. High-risk patients were indicative of poorer survival outcomes. The ROC curves confirmed the well predictive efficacy of this signature, which was superior to conventional clinical factors. The multivariate Cox analyses uncovered that IRS model was an independent risk factor of OSCC prognosis. More prospective cohorts should be adopted for verifying this prognostic model.

Secretion of HBEGF by M2 macrophages induces radioresistance of human papilloma virus-negative head and neck squamous cell carcinoma (HNSCC) (Fu et al., 2020). Moreover, HBEGF upregulation contributes to acquired cetuximab-resistance in HNSCC (Hatakeyama et al., 2010). HBEGF expression is in relation to HNSCC patients’ OS (Liu et al., 2020). HBEGF acts as an underlying regulator of invasion capacity of OSCC cells (Ohnishi et al., 2012). CCL22, mainly synthesized by M2 macrophages, contributes to a deterioration of clinical outcomes of patients with tongue SCC (Kimura et al., 2019). CCL22 downregulation in tongue and mouth floor SCC triggers reduced Th2 cell recruitment and expression and predicts undesirable survival outcomes (Li et al., 2021). Cancer-associated fibroblast-secreted IL-1β may activate CCL22 signaling in oral cancer (Huang et al., 2019). CTSG acts as an underlying immune-relevant marker in OSCC (Huang et al., 2021). PLAU facilitates cellular proliferation and epithelial–mesenchymal transition in HNSCC (Chen G. et al., 2021). This study nomogram incorporating the IRS model, stage, and age was established, which may enable clinicians to determine individual patient’s clinical outcomes. This graphical scoring system is easy to understand in developing the customized therapy as well as making medical decisions.

An integrative genomic analysis uncovers four main driver pathways (mitogenic signaling, notch, cell cycle, and TP53) as well as two additional somatic driver genes (FAT1 and CASP8) in OSCC (Pickering et al., 2013). Evidence suggests that high TMB displays an association with an undesirable survival outcome of HNSCC patients (Zhang et al., 2020). Herein, we noticed that high TMB patients exhibited significantly increased IRS than those with low TMB. Our subgroup analysis uncovered that IRS was predictive of OSCC prognosis independent of somatic mutations. Moreover, we noticed that cell cycle, oocyte meiosis, p53 signaling pathway, spliceosome, and ubiquitin-mediated proteolysis were markedly activated in high-risk patients, while arachidonic acid metabolism and primary immunodeficiency were significantly activated in low-risk patients. Thus, IRS was in relation to the activities of the above pathways during OSCC progression.

Our ssGSEA results uncovered the marked activity of immune activation pathways such as IL6-JAK-STAT3 signaling, allograft rejection, inflammatory response, complement, and IL2-STAT5 signaling in low-risk patients, while carcinogenic activation pathways such as TGF-beta signaling, PI3K-Akt-mTOR signaling, E2F targets, MYC targets, and mTORC1 signaling displayed enhanced activity in high-risk patients. Furthermore, decrease in stromal score, immune score, immune cell infiltration, and expression of HLA and immune checkpoints was found in high-risk specimens. Collectively, IRS might participate in shaping a nonflamed tumor microenvironment of OSCC. The cancer immunity cycle reflects the immune response to cancer (Chen and Mellman, 2017). The activity of each step in the cancer-immunity cycle determines the complex immunomodulatory interactions in the tumor microenvironment (Niu et al., 2022). Herein, we noticed that the IRS model displayed negative correlations with the activities of nearly all steps in the cancer immunity cycle including cancer antigen presentation, priming and activation, recruiting of B cell, CD4^+^ T cell, CD8^+^ T cell, dendritic cell, eosinophil, macrophage, MDSC, NK cell, T cell, Th1 cell, Th17 cell, Th2 cell, and Treg cell, infiltration of immune cells into tumors, recognition of cancer cells by T cells, and killing of cancer cells. In the anti-PD-L1 therapy cohort, this IRS model can predict patients’ clinical outcomes. Chemotherapeutic agents such as cisplatin, 5-fluorouracil, and paclitaxel have been the first-line therapeutic options for OSCC patients (Meng et al., 2021). However, most patients ultimately acquire drug resistance as well as undesirable clinical outcomes. Our IRS model possessed the potential in prediction of responses to methotrexate and paclitaxel in OSCC patients.

A few limitations of our study should be pointed out. Firstly, the sample size was relatively small. Secondly, though these findings were verified in an external cohort, more cohorts will be obtained for confirming our interesting findings. Thirdly, the optimal cutoff value of IRS was not determined. Herein, the median IRS was utilized as the cut-off. At last, more experiments will be presented for determining the biological significance of characteristic IRGs in this model.

## Conclusion

Collectively, our findings conducted an IRS model for OSCC and determined its clinical significance as a reliable biomarker in the prediction of patients’ prognosis and therapeutic benefits. Additionally, we uncovered the critical functions of IRGs on crosstalk between cancer cells and immune cells underlying oral carcinogenesis, eventually promoting the development of tailed immunotherapeutic strategies.

## Data Availability

The original contributions presented in the study are included in the article/Supplementary Material; further inquiries can be directed to the corresponding author.

## Abbreviations

AUC, area under the curve; C-index, concordance index; DFS, disease-free survival; CTLs, cytotoxic T lymphocytes; GDSC, Genomics of Drug Sensitivity in Cancer; ESTIMATE, Estimation of stromal and immune cells in malignant tumors using expression data; FPKM, fragments per kilobase million; GDC, Genomic Data Commons; GSEA, gene set enrichment analysis; GSVA, gene-set variation analysis; GEO, Gene Expression Omnibus; HLA, human leukocyte antigen; IRS, immune-related risk score; IC50, half-maximal inhibitory concentration; K-M, Kaplan–Meier; KEGG, Kyoto Encyclopedia of Genes and Genomes; MAF, mutation annotation format; MSigDB, Molecular Signatures Database; OSCC, oral squamous cell carcinoma; OS, overall survival; ROC, receiver operating characteristic; RNA-seq, RNA sequencing; ssGSEA, single-sample gene set enrichment analysis; TCGA, The Cancer Genome Atlas; TPM, transcripts per kilobase million; TMB, tumor mutation burden; IRGs, immune-related genes; TIDE, tumor immune dysfunction and exclusion.
